# Unveiling T cell evasion mechanisms to immune checkpoint inhibitors in acute myeloid leukemia

**DOI:** 10.20517/cdr.2023.39

**Published:** 2023-09-26

**Authors:** Lindsay Gurska, Kira Gritsman

**Affiliations:** ^1^Department of Cell Biology, Albert Einstein College of Medicine, Bronx, NY 10461, USA.; ^2^Department of Medical Oncology, Montefiore Medical Center, Albert Einstein College of Medicine, Bronx, NY 10461, USA.

**Keywords:** Acute myeloid leukemia, T cells, immune checkpoint, immune evasion

## Abstract

Acute myeloid leukemia (AML) is a heterogeneous and aggressive hematologic malignancy that is associated with a high relapse rate and poor prognosis. Despite advances in immunotherapies in solid tumors and other hematologic malignancies, AML has been particularly difficult to treat with immunotherapies, as their efficacy is limited by the ability of leukemic cells to evade T cell recognition. In this review, we discuss the common mechanisms of T cell evasion in AML: (1) increased expression of immune checkpoint molecules; (2) downregulation of antigen presentation molecules; (3) induction of T cell exhaustion; and (4) creation of an immunosuppressive environment through the increased frequency of regulatory T cells. We also review the clinical investigation of immune checkpoint inhibitors (ICIs) in AML. We discuss the limitations of ICIs, particularly in the context of T cell evasion mechanisms in AML, and we describe emerging strategies to overcome T cell evasion, including combination therapies. Finally, we provide an outlook on the future directions of immunotherapy research in AML, highlighting the need for a more comprehensive understanding of the complex interplay between AML cells and the immune system.

## INTRODUCTION

Acute myeloid leukemia (AML) is a devastating blood cancer and is the most common form of acute leukemia in adults. Long-term outcomes for AML have not significantly improved over the past few decades, with a suboptimal 5-year overall survival rate of 30% for AML patients ages 20 and older and less than 10% for AML patients ages 65 and older^[1]^. The current standard of care approaches for AML, including induction chemotherapy, combinations of venetoclax with hypomethylating agents, and stem cell transplantation, still yield high relapse rates with significant toxicities. Therefore, new less toxic therapeutic approaches need to be developed to improve survival and prevent relapse in this disease.

Hematopoiesis is the process through which all mature blood cell lineages are generated from hematopoietic stem cells (HSCs), which have the capacity to both self-renew and differentiate. Without proper regulation of their cell-intrinsic and cell-extrinsic cues (primarily signaling pathways, transcription factors, and epigenetic regulators), HSCs and downstream progenitors can acquire unlimited self-renewal potential at the expense of differentiation, as well as increased proliferation and survival, leading to AML development^[2-5]^. AML blasts develop from aberrant HSCs or progenitors - termed the leukemic stem cell (LSC). LSCs are undifferentiated blood cells that have pathologic self-renewal properties and lead to abnormal blood production. Phenotypically, LSCs share some of the same cell surface markers as HSCs, but unique LSC and pre-LSC gene expression signatures have been identified by high throughput sequencing^[6-9]^. Like HSCs, LSCs are primarily quiescent and are therefore resistant to chemotherapy and other therapies that target actively cycling cell populations^[3,10]^. Yet, the standard induction “7 + 3” chemotherapy regimen remains the preferred up-front treatment strategy for AML patients who are fit enough to tolerate intensive induction therapy, which, in addition to sparing LSCs, results in various toxicities, such as pancytopenia and infection^[11,12]^. This has led to enhanced research efforts to identify novel therapies that target the LSC population while sparing healthy HSCs to improve AML patient outcomes.

However, in addition to the cell-autonomous mechanisms AML cells have employed to persist despite the cytotoxic effects of chemotherapy, AML cells have developed additional ways to persist despite treatment, including resistance mechanisms to targeted therapies and immune evasion. Notably, AML cells employ several mechanisms, such as reliance on immune cells, to establish an immunosuppressive environment to ensure their survival. This is accomplished through the reduction of cytotoxic and effector T and NK cells, increased T cell exhaustion, and recruitment of immunosuppressive populations such as regulatory T cells, myeloid-derived suppressor cells (MDSCs), and M2 macrophages^[13]^. Importantly, it has been reported that the number of effector and cytotoxic T cells, termed tumor-infiltrating lymphocytes (TILs), present in the bone marrow can be a prognostic marker for overall survival and leukemia-free survival^[14]^. In addition, increased numbers and function of regulatory T cells in both the peripheral blood and bone marrow of AML patients have been reported, with bone marrow-resident regulatory T cells exhibiting more immunosuppressive effects on CD4+ effector T cell proliferation^[15]^. A lower frequency of regulatory T cells was found to correlate with complete remission rates in AML patients, while a higher frequency was observed in patients who relapsed^[15]^.

Despite advances in immunotherapies in solid tumors and some lymphoid malignancies, AML has been particularly difficult to treat with immunotherapies, primarily due to poor T cell recruitment to the bone marrow and because LSCs are immune privileged. Because even with the current therapeutic options, AML remains a lethal disease with a suboptimal long-term survival rate, it is imperative to identify and exploit the mechanisms by which AML cells evade immune detection to unleash the potential benefits of immunotherapy in AML treatment. This review summarizes the roles of T cells in the immune response, and highlights the challenges that AML cells pose to the efficacy of ICIs by evading T cell detection.

## T CELLS FRONT THE ADAPTIVE IMMUNE RESPONSE

The adaptive immune system plays an essential role in eliminating a variety of threats to our bodies, including cancer and infection. Key players in the adaptive immune response are B lymphocytes (B-cells) and T lymphocytes (T cells). They are distinguishable from cell types that primarily function in the innate immune response because they have antigen-specific receptors - B-cell receptor (BCR) and T cell receptor (TCR), respectively^[16]^. T cells can differentiate into three different cell types: effector T cells, cytotoxic T cells, and regulatory T cells. Effector T cells, also known as “helper T cells”, which express the cell-surface protein CD4, function through cytokine signaling, such as interferon gamma (IFNγ) and tumor necrosis factor alpha (TNFα), which stimulate other immune cells^[16]^. Cytotoxic T cells, which express the cell-surface protein CD8, program invading cells to undergo apoptosis via the secretion of granzyme B, perforin, and IFNγ^[16]^. Unlike effector and cytotoxic T cells, regulatory T cells function to suppress immune cells to mitigate any possible damage from a prolonged immune response, and to prevent auto-immunity^[16]^. They can be identified through flow cytometry by the expression of CD4, CD25, and FoxP3^[16]^.

**Figure 1:**
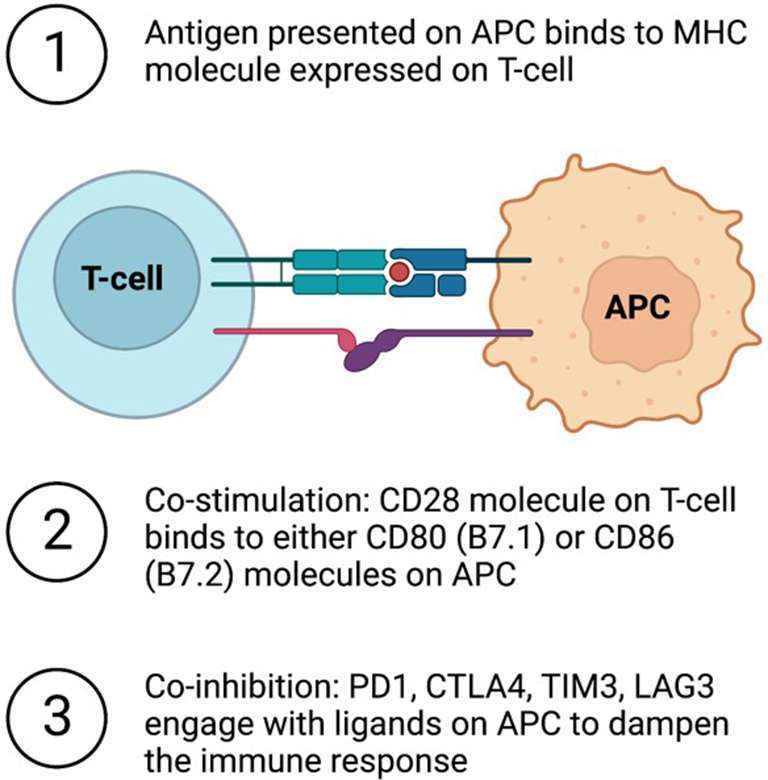
T cell-mediated immune response. Overview of the steps required for full T cell activation. Figure created with Biorender.com. APC: Antigen-presenting cell; CTLA4: cytotoxic T lymphocyte antigen 4; LAG3: lymphocyte activation gene-3; MHC: major histocompatibility complex; PD1: programmed cell death protein 1; TIM3: T cell immunoglobulin and mucin domain-containing protein 3.

In order to activate a T cell-mediated immune response, two different signals are required. The first signal occurs when the disease-causing cell presents an antigen, or host-derived protein molecule, to a T cell [Figure 1]. Specifically, short peptide fragments of an antigen are presented on the surface of host cells, termed antigen-presenting cells (APCs), by major histocompatibility complex (MHC) molecules. There are two classes of MHC molecules, MHC class I and MHC class II. Notably, CD8+ T cells selectively recognize MHC class I molecules, while CD4+ T cells selectively recognize MHC class II molecules. MHC class II molecules are often expressed on dendritic cells and macrophages, which engulf the antigen and process it for presentation. MHC class II molecules can also be present on the surface of foreign APCs. The Class II transactivator (CIITA) is a master regulator of MHC gene expression^[17]^. CIITA responds to IFNγ activation, where it then acts as a transcriptional activator to turn on MHC gene expression^[17]^.

The second signal required for T cell activation is termed the co-stimulatory signal, and co-stimulation is thought to occur through the interaction between the CD28 molecule on T-lymphocytes and either CD80 (B7.1) or CD86 (B7.2) molecules on the APC^[18]^ [Figure 1]. The discovery of CD28 and its essential role in T cell activation has led to further discovery of other cell-surface molecules that regulate T cell activity. Interestingly, the discovery of cytotoxic T lymphocyte antigen 4 (CTLA4) on T cells identified another binding partner of B7-1. However, CTLA4 expression is induced following T cell activation, where it can out-compete CD28 binding to B7.1 to dampen the T cell response^[18]^.

This has led to the discovery and categorization of other cell-surface molecules that positively (referred to as co-stimulatory receptors) and negatively (co-inhibitory receptors) modulate T cell activity. Other co-inhibitory receptors on T cells include programmed cell death protein 1 (PD1), which binds to its ligands programmed death-ligand 1 (PD-L1, also known as B7-H1) or programmed death-ligand 2 (PD-L2, also known as B7-H2) on APCs; T cell immunoglobulin and mucin domain-containing protein 3 (TIM3), and lymphocyte activation gene-3 (LAG3)^[19,20]^. TIM3 binds to various ligands (including Galectin-9, Ceacam-1, and HMGB-1), while LAG3 binds to MHC class II molecules with higher affinity than the CD4+ TCR^[19,20]^. Other co-inhibitory ligands on APCs include B7-H3, B7-H4, and B7-H5^[21]^.

## MECHANISMS OF IMMUNE EVASION IN AML

**Figure 2:**
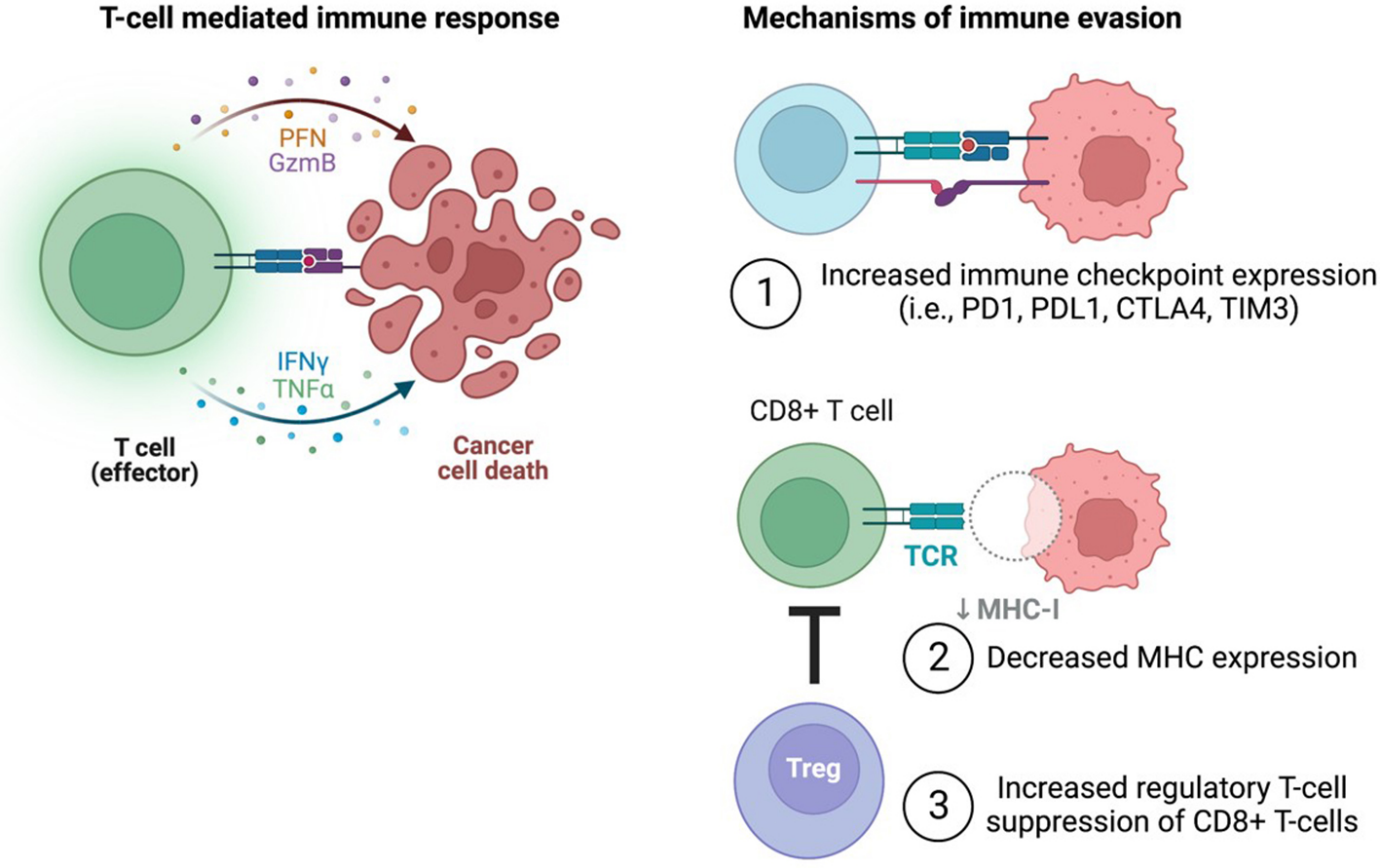
Mechanisms of T cell evasion in AML. T cells engage with and kill cancer cells through the presentation of MHC molecules and subsequent T cell co-stimulation (left). Mechanisms to evade T cell detection employed by AML cells include (1) increased expression of co-inhibitory immune checkpoints; (2) decreased MHC expression; and (3) suppression of cytotoxic CD8+ T cell function through increased regulatory T cells (right). Figure created with Biorender.com. AML: Acute myeloid leukemia; CTLA4: cytotoxic T lymphocyte antigen 4; IFNγ: interferon gamma; MHC: major histocompatibility complex; PD1: programmed cell death protein 1; PFN: perforin; TCR: T cell receptor; TIM3: T cell immunoglobulin and mucin domain-containing protein 3; TNFα: tumor necrosis factor alpha.

There are currently several different immunotherapy strategies being investigated in hematologic malignancies, including in AML^[11,21]^. Immune checkpoint inhibitors, such as antibodies targeting CTLA4 and PD1, have been approved for the treatment of some types of lymphoma and some solid tumors, including melanoma, lung cancer, kidney cancer, head and neck cancer, bladder cancer, and colorectal cancer^[22]^. However, in AML, the use of immune checkpoint inhibitors has been more challenging, and there are no FDA approvals of this class of agents in AML to date. This is in part due to the various cell-autonomous and cell non-autonomous mechanisms that leukemic cells employ to reprogram themselves and the bone marrow microenvironment to render them immune privileged [Figure 2]. Additionally, ICIs are often most effective in cancers with a high mutation burden (i.e., melanoma, lung cancer), which is often not as high in AML^[23]^. For example, many AML patients have a defined blast population with 1-2 driver mutations and/or cytogenetic alterations, with sub-clones that may not arise until disease progression or relapse^[24]^.

### Increased immune checkpoint expression

Immune checkpoints are known to be a key mechanism that mediates T cell immunosuppression in AML. Interesting work using PD1 knockout mice delineated the importance of this axis in regulatory T cells and CD8+ cytotoxic T cells. Specifically, AML development was impeded when AML cells were injected into PD1 knockout mice^[25]^. This was dependent on the ability of regulatory T cells to suppress CD8+ T cells via enhanced PD1 expression on T cells and PD-L1 expression on APCs^[25]^. Interestingly, treating mice that developed AML with IL-2 linked to diphtheria toxin (IL-2DT), followed by anti-PDL1 monoclonal antibody treatment, markedly reduced the AML tumor burden^[25]^. Therefore, this work suggests that strategies to deplete regulatory T cells and inhibit the PD1/PD-L1 interaction could be effective in overcoming the AML-privileged microenvironment.

T cell exhaustion is also a mechanism for immune evasion and is often phenotypically characterized by the expression of the immune checkpoint TIM3. In one study evaluating the role of exhausted T cells in AML relapse following transplantation, the frequency of PD1-high TIM3-positive T cells was significantly correlated with AML relapse^[26]^. These T cells were confirmed to be exhausted, as they exhibited reduced production of IL-2, TNFα, and IFNγ^[26]^. The impact of this study was clinically significant, as the expression of exhaustion markers on T cells could be detected before the diagnosis of relapse^[26]^. These results were echoed in a subsequent study that characterized the exhausted T cell population following AML relapse post-transplantation, which, despite displaying specific leukemic blast recognition (determined by CDR3 sequencing of TCR-α and TCR-β chains), had impaired effector T cell function^[27]^. As the prognosis for patients who relapse after transplantation is poor, early detection of T cell exhaustion markers could be a useful predictive tool^[26,27]^.

Modulation of checkpoint expression on AML cells themselves is another key driver of immune evasion. For example, increased PD-L1, PD-L2, and CTLA4 expression on AML cells has been shown to correlate with poor overall survival^[28,29]^. PD-L1 expression was found to be elevated in AML patient blasts, both at diagnosis and at relapse^[30]^. Furthermore, CTLA4 was previously discovered to not be restricted to the lymphoid lineage, as AML cells from both diagnostic and relapsed patients, but not healthy CD34+ cells, were found to express CTLA4^[31,32]^. Therefore, the upregulation of inhibitory immune checkpoints on AML cells is another potential mechanism for immune evasion in AML.

### Downregulation of MHC expression

Dampening of MHC expression on AML cells is also an important mechanism of immune evasion. Specifically, RNA sequencing analysis of paired AML samples collected at diagnosis and at relapse post-transplantation identified altered expression of immune-related genes, including decreased expression of CIITA, the master regulator of MHC-II expression, and of MHC-II molecules at relapse^[33]^. *Ex vivo* treatment of AML blasts isolated from relapse patients with IFNγ was able to restore MHC-II expression^[33]^. The clinical significance of this is revealed by the differences in CD4+ effector T cell activation, as measured by IFNγ production, following co-culture of either diagnostic or post-transplantation relapsed AML samples with CD4+ T cells, as CD4+ T cell activity was diminished in post-transplantation relapse co-culture assays^[33]^.

Accordingly, in a recent transcriptome analysis of AML cells from patients who relapsed following transplantation, a transcription factor complex consisting of IRF8, MYB, MEF2C, and MEIS1 was found to regulate MHC expression in AML, and combinatorial changes in their expression are essential for reduced MHC expression at relapse^[34]^. Interestingly, the authors found a small cell population with silenced MHC expression at leukemia diagnosis, and concluded that, similar to resistant LSC populations, this population may be selected after transplantation and can contribute to relapse^[34]^. Overall, these mechanisms are plausible explanations for why the treatment of patients who relapse post-transplantation is particularly challenging. Identifying ways to overcome decreased MHC expression following transplantation is underway. For example, a recent study using AML xenograft mouse models reported that MDM2 inhibition can increase MHC-II production, and CD8+ T cells isolated from MDM2 inhibitor-treated primary AML mice can eradicate disease in secondary recipients^[35]^.

### The role of regulatory T cells in the immunosuppressive microenvironment

The increased number and activity of regulatory T cells (Tregs) in the leukemic bone marrow renders the bone marrow an immunosuppressive microenvironment due to their effects on effector and cytotoxic T cell populations. Recent insights have identified mechanisms for increased Treg function in the AML microenvironment, such as via increased expression and production of IFNγ by AML cells, leading to upregulation of genes that promote differentiation into Tregs^[36]^. Recognizing the correlation between increased Treg numbers and poor AML outcomes, one group investigated the effects of Treg ablation on leukemogenesis^[37]^. Using Foxp3-DTR to ablate Tregs in mice, they observed prolonged survival in MLL-AF9-induced AML mouse models and increased CD8+ T cell activity^[37]^. As Treg ablation is likely not easily transferrable to the clinic, they also identified additional ways to impede Treg accumulation in the leukemic microenvironment in mice, including CCL3-CCR1/CCR5 and CXCL12-CXCR4 blockade^[37]^. Importantly, as increased regulatory T cell populations are also a predictor of AML relapse following transplantation^[27]^, it is critical to exploit mechanisms that decrease Treg numbers and function.

### NK cell-mediated immune evasion mechanisms

AML cells can also evade detection by NK cells, which are canonically activated by the recognition of stress-induced ligands on foreign cells^[38]^. Similar to their evasion of T cells, AML cells can also evade NK-cell recognition and elimination through multiple mechanisms, including (1) the reduced expression of stress-induced ligands on AML cells; (2) increased expression of inhibitory receptors on NK cells to suppress NK cell function; (3) the induction of the immunosuppressive environment to limit NK cell numbers and function; and (4) activation of anti-apoptotic pathways to resist NK-cell induced cell death^[39,40]^. These NK-cell evasion mechanisms, as well as strategies to target them, have been extensively reviewed elsewhere^[39,41-44]^. For example, it was shown that epigenetic mechanisms mediate the silencing of NKG2D ligands in AML, and that treatment with hypomethylating agents can increase their expression and subsequent NK-cell recognition^[45]^. Furthermore, pivotal work demonstrated that LSCs are immune privileged through their lack of expression of NKG2D ligand, which is essential for NK-cell detection and subsequent clearance^[46]^. As NK2GD remains a hot target for immunotherapy in AML^[47-49]^, it is important to appreciate that other mechanisms may be required to eliminate the LSC population.

Another mechanism that can mediate NK-cell evasion is CD48 silencing^[50-52]^. It was demonstrated that high CD48 expression on AML cells is correlated with a favorable prognosis. However, in a subset of AML patients, CD48 expression can be suppressed through enhanced methylation^[52]^. Therefore, treatment with hypomethylating agents may be able to increase CD48 expression to increase NK-cell killing^[53]^. Overall, understanding NK-cell evasion mechanisms is critical to overcoming immunotherapy resistance and identifying targets for immunotherapy.

## CLINICAL INVESTIGATION OF IMMUNE CHECKPOINT INHIBITORS IN AML

Several strategies that incorporate checkpoint inhibitors have been tested in AML in clinical trials, and several more clinical trials are underway [Table 1]. In a phase 1/1b clinical trial of ipilimumab (anti-CTLA4) in patients with hematologic malignancies that relapsed after allogeneic stem cell transplantation, analysis of the AML subset (12/28 patients) showed that 5/12 patients achieved complete remission following treatment, and this was accompanied by a reduction in the frequency of circulating Tregs compared to non-responders^[54]^.

**Table 1:** Overview of ongoing clinical trials of immune checkpoint inhibitors in AML

**Target**	**Agent**	**Regimen**	**Population**	**Phase**	**NCT identifier**	**Primary endpoints**
PD-1	Pembrolizumab	IC ± Pembrolizumab	ND AML	2	NCT04214249 (BLAST MRD AML-1)	MRD-CR
		VEN + AZA ± Pembrolizumab	ND AML	2	NCT04284787 (BLAST MRD AML-2)	MRD-CR
		HiDAC followed by Pembrolizumab	R/R AML	2	NCT02768792	CR
		Decitabine + Pembrolizumab ± VEN	ND or R/R AML	1	NCT03969446	Incidence of AE, MTD, CR
	Nivolumab	Nivolumab	AML patients in remission after IC	2	NCT02275533 (REMAIN TRIAL)	PFS
		Nivolumab	AML patients in remission after IC	2	NCT02532231	Recurrence-free survival
		AZA + Nivolumab ± Ipilimumab	ND or R/R AML	2	NCT02397720	MTD, ORR
		Decitabine + VEN + Pembrolizumab	ND *TP53-*mutant AML	1	NCT04277442	Incidence of AE, CR
		Nivolumab ± Ipilimumab	AML patients post-HSCT	1	NCT03600155	Optimal dose
CTLA-4	Ipilimumab	Decitabine + Ipilimumab	R/R AML	1	NCT02890329	MTD
		Ipilimumab + CD25hi Treg-depleted DLI	R/R AML post-HSCT	1	NCT03912064	MTD

AE: Adverse event; AML: acute myeloid leukemia; AZA: Azacitidine; CR: complete remission; DLI: donor lymphocyte infusion; HSCT: hematopoietic stem cell transplant; HiDAC: high dose cytarabine; IC: intensive chemotherapy; MRD-CR: minimal residual disease negative complete remission; MTD: maximum-tolerated dose; ND: newly diagnosed; ORR: overall response rate; PFS: progression-free survival; R/R: relapsed/refractory; VEN: Venetoclax. Source: clinicaltrials.gov.

Furthermore, in a Phase II study investigating the combination of high-dose cytarabine and pembrolizumab (anti-PD1) in relapsed/refractory AML patients, 14 out of 37 patients achieved complete remission (CR). Interestingly, of the patients that achieved a CR, TCR signaling identified a trend towards increased TCR diversity in these patients, as well as decreased regulatory T cell and increased CD8+ T cell frequencies^[55]^. Of note, RNA-seq analysis of AML blasts from these patients revealed that increased MHC expression was significantly upregulated at baseline in patients who achieved CR compared to non-responders^[55]^.

Interestingly, recent data suggests that PD1 signaling may be implicated in the poor response to hypomethylating agents (HMAs), including azacitidine and decitabine, as patients who are resistant to HMAs show higher expression of PD-L1, PD-L2, and CTLA4^[56-58]^. On the other hand, preclinical findings from single-agent immune checkpoint inhibitor trials in AML have demonstrated limited efficacy. This has prompted the investigation of checkpoint inhibitors in combination with HMAs^[57]^. In a phase 1b trial investigating the combination of ipilimumab with decitabine in relapsed/refractory AML, patients who were transplant naïve (*N* = 23) observed a higher response rate than those who relapsed following stem cell transplantation (*N* = 20) (CR + CRi + mCR 52% *vs.* 20%, *P* = 0.034; median overall survival 16.2 months *vs.* 8.6 months)^[59]^. Not surprisingly, when performing integrative transcriptome-based analysis of bone marrow infiltrating cells from participating patients, a high baseline ratio of T cells to AML cells was associated with higher response rates^[60]^. The authors speculated that the inadequate clearance of the immature LSC population triggered relapse in patients following stem cell transplantation, but also noted that ipilimumab exposure resulted in increased memory T cell bone marrow infiltration and high expression of CTLA4 and FOXP3, suggesting that the efficacy of ipilimumab and decitabine may be impacted by these immune evasion mechanisms employed by LSCs^[60]^. The results of the ipilimumab and decitabine combination studies also highlight the limitations of ICIs in AML. A comparison of the memory and exhaustion gene scores associated with CD8+ T cells from AML bone marrow with those from CD8+ TILs isolated from solid tumors, in which ipilimumab demonstrates high clinical activity, revealed higher exhaustion profiles and checkpoint expression in solid tumor-derived T cells^[60]^.

In two ongoing trials testing the combination of pembrolizumab and decitabine in relapsed/refractory AML, interim results showed a tolerable safety profile with promising efficacy data^[56,61]^. Furthermore, through the generation of RNA expression datasets from patients who were treated with conventional cytotoxic chemotherapy or with pembrolizumab and azacitidine in relapsed/refractory AML, Rutella *et al.* revealed a newly defined CD8+ T cell senescent gene population with a distinct gene expression signature^[62]^. These cells were impaired in their ability to kill AML blasts isolated from the same patient sample, and their frequency negatively correlated with overall survival^[62]^. However, there is still promise for the combination of PD1 blockade and HMA, as results from the Phase II trial investigating nivolumab and azacitidine in relapsed/refractory AML yielded a 33% overall response rate, with a higher response rate in HMA naïve *vs.* HMA pre-treated patients (58% *vs.* 22%)^[63]^. Based on these clinical trials, the possible predictors of response to immune checkpoint inhibitors are summarized in Table 2. Overall, given these data, the field is anxiously awaiting the results of additional clinical trials currently that are investigating immune checkpoint inhibitors in AML.

**Table 2:** Possible predictors of response to immune checkpoint inhibitors in AML or MDS

**Immune checkpoint inhibitor**	**Clinical setting**	**Possible predictors of response**	**Response assessment**	**Ref**
Ipilimumab	Post-HSCT	-Baseline donor T cell chimerism of > 99% -Lower frequency of CD4+ Tregs -Increase in plasma CXCL2, CXLC5, CXCL6, IL1R, ANGPT-1 and -2, VEGF	CR or stable disease	[54]
Pembrolizumab	R/R AML, post-HiDAC	-Trend towards higher TCR diversity at baseline -Higher frequency of senescent T cells in BM -Higher frequency of terminally differentiated effector T cells in PB -Increased frequency of CD8+ T cells expressing CD28, PD-1, and TIGIT in BM -Presence of pre-treatment CD8+ T cells co-expressing TCF-1 and PD-1 -Transcriptional upregulation of PI3K/AKT/MTOR signaling pathway in BM blasts	CR	[55]
Ipilimumab	In combination with decitabine in AML or MDS before and after HSCT	-No clear predictors of response	Leukemic cell burden, frequency of infiltrating lymphocytes	[59]
Ipilimumab	In combination with decitabine in AML or MDS before and after HSCT	-Lower VAF of recurrent AML/MDS-associated mutations -Higher T cell to AML ratio -Increased T cell to myeloid ratio -Donor-derived myeloid cells present at higher % in responders -Higher circulating expression of CCL17, CXCL1, CXCL5, EGF, LAMP3, and PDGF subunit B	CR/CRi	[60]
Pembrolizumab	In combination with decitabine in R/R AML	-Trend towards increased CD3+ infiltrates in BM during treatment -No association of TCRb sample clonality with response	CR	[61]
Pembrolizumab	In combination with azacitidine in newly diagnosed AML *vs.* cytotoxic chemotherapy	-Increased proportion of CD3+CD8+CD57+KLRG1+ senescent T cells in baseline BM associated with worse OS -Increased proportion of senescent T cells in BM post-treatment associated with worse OS -High IED signature score associated with worse OS	OS	[62]
Nivolumab	In combination with azacytidine in R/R AML	-Trend towards association with improved response: no prior HMA, presence of ASXL1 mutation -Higher frequency of pre-treatment BM % CD3+ T cells in responders -Trend towards higher frequency of CD4+ T effector cells and CD8+ T cells in pre-treatment BM in responders	ORR	[63]

AML: Acute myeloid leukemia; BM: bone marrow; CR: complete remission; CRi: incomplete remission; HiDAC: high-dose cytarabine; HMA: hypomethylating agent; HSCT: hematopoietic stem cell transplantation; IED: immune effector dysfunction; MDS: myelodysplastic syndrome; ORR: overall response rate; OS: overall survival; PB: peripheral blood; R/R AML: relapsed or refractory acute myeloid leukemia; TCR: T cell receptor; Tregs: regulatory T cells; VAF: variant allele frequency.

Further investigation into the molecular mechanisms that both AML cells and T cells employ to evade immune detection may help to identify novel combination strategies for ICIs in AML. For example, altered signaling and expression of cellular proteins due to genetic alterations are hallmarks of AML cells. With both approved and investigational therapies available to target oncogenes (e.g., FLT3, IDH1/2, NPM1c/Menin inhibitors) responsible for regulating the expression and/or post-translational modifications (e.g., methylation, acetylation, glycosylation, ubiquitination) of proteins in AML cells, it is critical to determine if targetable driver mutations are important for the increased expression of immune checkpoints in AML cells.

Alternatively, further investigation into the mechanisms that T cells employ to increase checkpoint expression or to increase Treg function is warranted to improve ICI outcomes in AML. For example, a recent study analyzing the transcriptome of CD8 T cells from the bone marrow of AML patients demonstrated the downregulation of genes responsible for T cell activation, differentiation, and function (e.g., NF-KB, FOXO, cytokine/chemokine signaling)^[64]^. With several of these genes being involved in epigenetic regulation, the authors postulate that epigenetic changes to T cells may impair TCR activation and overall T cell function^[64]^. However, additional studies are necessary.

Lastly, additional studies are underway to identify mechanisms that increase the frequency of Tregs, with some insights regarding tumor necrosis factor receptor-2 (TNFR2) and the TNFα pathway playing an important role in increasing the frequency of Tregs in AML patient samples^[65]^, in addition to increased expression of IFNγ via *IDO1* overexpression in mesenchymal stem cells^[36]^. Importantly, the mechanisms employed by AML cells and T cells may be interrelated, as suggested by recent evidence collected in AML cell lines that induced expression of PD-L1 on AML cells could result in the conversion and subsequent expansion of CD4+CD25+FoxP3+ Tregs from CD4+ T cells^[66]^.

## CONCLUSION

In summary, through antigen recognition and co-stimulation, T cells front the adaptive immune response, causing AML cells to employ both cell-autonomous and cell non-autonomous mechanisms to create an immunosuppressive microenvironment and evade detection and killing by T cells. These mechanisms include (1) reduced expression of antigens and MHC molecules on the cell surface of AML cells; (2) immune checkpoint activation to suppress T cell responses, both on T cells and on AML cells themselves; (3) induction of T cell exhaustion; and (4) the induction of an immunosuppressive environment by increasing the numbers of regulatory T cells and other immunosuppressive populations in the bone marrow to inhibit effector and cytotoxic T cell activity. All of these mechanisms ultimately promote AML cell survival. This review complements several other recent review articles in this field, which illustrate the importance of understanding the mechanisms of immune evasion in AML to overcome immunotherapy resistance and improve AML outcomes^[13,53,67-69]^.

In our review of the current ICI landscape for hematologic malignancies, evident frustrations arise when comparing the success of checkpoint inhibitors in solid tumors to the more limited progress made with these agents in AML. The mechanisms highlighted above undoubtedly contribute to the slow adoption of ICIs in AML. With many clinical trials underway in this space, continued research efforts identifying ways to overcome immunotherapy resistance, such as combining ICIs with targeted therapies against components of signaling pathways notoriously activated in AML, as seen in solid tumors^[70]^, are warranted. Furthermore, while not a major focus of this review, it remains a challenge to identify tumor-specific targets for personalized immunotherapies for AML, such as CAR T cells and bispecific antibodies^[71-73]^.

While this review provides some insights into the roles of immune evasion mechanisms in relapse following stem cell transplantation, as well as the clinical trials underway utilizing ICIs for this patient population, the poor prognosis rates for AML patients who relapse after transplantation highlight the need for a review focused on this specifically. Some groups have taken this initiative already, including a summary of the current understanding of the downregulation of HLA molecules and inhibitory checkpoints between T cells and AML cells^[74]^. Additionally, recent insights into novel mechanisms by which an altered immune landscape following transplantation – characterized by increased expression of TIGIT and CD161 within the CD4+ T cell population post-transplantation – has begun to identify predictors of relapse^[75]^. A more recent review focuses on epigenetic mechanisms that underlie T cell evasion in the relapse post-transplant setting, and is also a good source for this topic^[76]^.

Lastly, this review does not cover the advances and limitations of emerging immunotherapy treatment modalities in AML- notably chimeric antigen receptor (CAR) T- and NK-cell therapies, bispecific antibodies, dual affinity re-targeting (DART) molecules, monoclonal antibodies, and antibody-drug conjugates. While these agents are approved in other cancers [e.g., acute lymphocytic leukemia (ALL), non-Hodgkin lymphoma (NHL) subtypes, and multiple myeloma (MM)], their adoption in AML has been slow, due to the difficulty of finding AML-specific antigens that are not also expressed on HSCs or myeloid progenitors. Furthermore, mechanisms of antigen escape, the AML immunosuppressive environment, and the impaired quality of autologous cells are also potential problems with these approaches, as reviewed elsewhere^[72]^. Nonetheless, current clinical trials underway in relapsed/refractory AML include CD33, CD38, CD123, and CD19 CAR-T cell therapies, allogenic CAR NK-cells, and CD33xCD3 and CD123xCD3 bispecific antibodies^[11,41]^.

Overall, as we continue to uncover the mechanisms underlying immune evasion in AML, exploiting these mechanisms will be of high priority to unleash the potential of immunotherapy in this disease. This is exemplified by the pivotal work done already, identifying a niche for immune checkpoint inhibitors after observing increased checkpoint expression in AML cells following HMA treatment^[58]^. Additionally, it will be important to identify strategies to suppress regulatory T cell activity in AML to allow for the unleashing of effector and cytotoxic and T cell activity. Thinking ahead, continued efforts to identify patient populations at higher risk for immune evasion during available treatments or following stem cell transplantation, such as characterizing TIL populations prior to and during treatment, or examining T cell and NK cell numbers and function in specific molecular or cytogenetic subgroups of AML will pave the way for more personalized AML treatment plans.

## DECLARATIONS

### Copyright

© The Author(s) 2023.

## References

[1] https://www.cancer.org/research/cancer-facts-statistics/all-cancer-facts-figures/2023-cancer-facts-figures.html.

[2] Shlush LI, Feldman T (2021). The evolution of leukaemia from pre-leukaemic and leukaemic stem cells. J Intern Med.

[3] Pollyea DA, Jordan CT (2017). Therapeutic targeting of acute myeloid leukemia stem cells. Blood.

[4] Corces-Zimmerman MR, Hong WJ, Weissman IL, Medeiros BC, Majeti R (2014). Preleukemic mutations in human acute myeloid leukemia affect epigenetic regulators and persist in remission. Proc Natl Acad Sci U S A.

[5] Gurska LM, Ames K, Gritsman K (2019). Signaling pathways in leukemic stem cells. Adv Exp Med Biol.

[6] Velten L, Story BA, Hernández-Malmierca P (2021). Identification of leukemic and pre-leukemic stem cells by clonal tracking from single-cell transcriptomics. Nat Commun.

[7] Ng SW, Mitchell A, Kennedy JA (2016). A 17-gene stemness score for rapid determination of risk in acute leukaemia. Nature.

[8] van Galen P, Hovestadt V, Wadsworth Ii MH (2019). Single-cell RNA-Seq reveals AML hierarchies relevant to disease progression and immunity. Cell.

[9] Eppert K, Takenaka K, Lechman ER (2011). Stem cell gene expression programs influence clinical outcome in human leukemia. Nat Med.

[10] Pandolfi A, Barreyro L, Steidl U (2013). Concise review: preleukemic stem cells: molecular biology and clinical implications of the precursors to leukemia stem cells. Stem Cells Transl Med.

[11] Newell LF, Cook RJ (2021). Advances in acute myeloid leukemia. BMJ.

[12] Tallman MS, Wang ES, Altman JK (2019). Acute myeloid leukemia, version 3.2019, NCCN clinical practice guidelines in oncology. J Natl Compr Canc Netw.

[13] Vago L, Gojo I (2020). Immune escape and immunotherapy of acute myeloid leukemia. J Clin Invest.

[14] Ismail MM, Abdulateef NAB (2017). Bone marrow T-cell percentage: a novel prognostic indicator in acute myeloid leukemia. Int J Hematol.

[15] Zhang S, Han Y, Wu J (2011). Elevated frequencies of CD4^+^CD25^+^CD127^lo^ regulatory T cells is associated to poor prognosis in patients with acute myeloid leukemia. Int J Cancer.

[16] Murphy K, Weaver C https://inmunologos.files.wordpress.com/2020/08/janeways-immunobiology-9th-ed_booksmedicos.org_.pdf.

[17] Devaiah BN, Singer DS (2013). CIITA and its dual roles in MHC gene transcription. Front Immunol.

[18] Chen L, Flies DB (2013). Molecular mechanisms of T cell co-stimulation and co-inhibition. Nat Rev Immunol.

[19] Anderson AC, Joller N, Kuchroo VK (2016). Lag-3, Tim-3, and TIGIT: co-inhibitory receptors with specialized functions in immune regulation. Immunity.

[20] Giannopoulos K (2019). Targeting immune signaling checkpoints in acute myeloid leukemia. J Clin Med.

[21] Wang H, Kaur G, Sankin AI, Chen F, Guan F, Zang X (2019). Immune checkpoint blockade and CAR-T cell therapy in hematologic malignancies. J Hematol Oncol.

[22] Lamble AJ, Lind EF (2018). Targeting the immune microenvironment in acute myeloid leukemia: a focus on T cell immunity. Front Oncol.

[23] Klempner SJ, Fabrizio D, Bane S (2020). Tumor mutational burden as a predictive biomarker for response to immune checkpoint inhibitors: a review of current evidence. Oncologist.

[24] Chen J, Kao YR, Sun D (2019). Myelodysplastic syndrome progression to acute myeloid leukemia at the stem cell level. Nat Med.

[25] Zhou Q, Munger ME, Highfill SL (2010). Program death-1 signaling and regulatory T cells collaborate to resist the function of adoptively transferred cytotoxic T lymphocytes in advanced acute myeloid leukemia. Blood.

[26] Kong Y, Zhang J, Claxton DF (2015). PD-1^hi^TIM-3^+^ T cells associate with and predict leukemia relapse in AML patients post allogeneic stem cell transplantation. Blood Cancer J.

[27] Noviello M, Manfredi F, Ruggiero E (2019). Bone marrow central memory and memory stem T-cell exhaustion in AML patients relapsing after HSCT. Nat Commun.

[28] Chen C, Liang C, Wang S (2020). Expression patterns of immune checkpoints in acute myeloid leukemia. J Hematol Oncol.

[29] Brodská B, Otevřelová P, Šálek C, Fuchs O, Gašová Z, Kuželová K (2019). High PD-L1 expression predicts for worse outcome of leukemia patients with concomitant *NPM1* and *FLT3* mutations. Int J Mol Sci.

[30] Berthon C, Driss V, Liu J (2010). In acute myeloid leukemia, B7-H1 (PD-L1) protection of blasts from cytotoxic T cells is induced by TLR ligands and interferon-gamma and can be reversed using MEK inhibitors. Cancer Immunol Immunother.

[31] Pistillo MP, Tazzari PL, Palmisano GL (2003). CTLA-4 is not restricted to the lymphoid cell lineage and can function as a target molecule for apoptosis induction of leukemic cells. Blood.

[32] Laurent S, Palmisano GL, Martelli AM (2007). CTLA-4 expressed by chemoresistant, as well as untreated, myeloid leukaemia cells can be targeted with ligands to induce apoptosis. Br J Haematol.

[33] Christopher MJ, Petti AA, Rettig MP (2018). Immune escape of relapsed AML cells after allogeneic transplantation. N Engl J Med.

[34] Eagle K, Harada T, Kalfon J (2022). Transcriptional plasticity drives leukemia immune escape. Blood Cancer Discov.

[35] Ho JNHG, Schmidt D, Lowinus T (2022). Targeting MDM2 enhances antileukemia immunity after allogeneic transplantation via MHC-II and TRAIL-R1/2 upregulation. Blood.

[36] Corradi G, Bassani B, Simonetti G (2022). Release of IFNγ by acute myeloid leukemia cells remodels bone marrow immune microenvironment by inducing regulatory T cells. Clin Cancer Res.

[37] Wang R, Feng W, Wang H (2020). Blocking migration of regulatory T cells to leukemic hematopoietic microenvironment delays disease progression in mouse leukemia model. Cancer Lett.

[38] Chan CJ, Smyth MJ, Martinet L (2014). Molecular mechanisms of natural killer cell activation in response to cellular stress. Cell Death Differ.

[39] Lion E, Willemen Y, Berneman ZN, Van Tendeloo VF, Smits EL (2012). Natural killer cell immune escape in acute myeloid leukemia. Leukemia.

[40] Khaznadar Z, Boissel N, Agaugué S (2015). Defective NK cells in acute myeloid leukemia patients at diagnosis are associated with blast transcriptional signatures of immune evasion. J Immunol.

[41] Xu J, Niu T (2020). Natural killer cell-based immunotherapy for acute myeloid leukemia. J Hematol Oncol.

[42] Carlsten M, Järås M (2019). Natural killer cells in myeloid malignancies: immune surveillance, NK cell dysfunction, and pharmacological opportunities to bolster the endogenous NK cells. Front Immunol.

[43] Kaweme NM, Zhou F (2021). Optimizing NK cell-based immunotherapy in myeloid leukemia: abrogating an immunosuppressive microenvironment. Front Immunol.

[44] (2019). Raneros A, López-Larrea C, Suárez-Álvarez B. Acute myeloid leukemia and NK cells: two warriors confront each other. Oncoimmunology.

[45] Baragaño Raneros A, Martín-Palanco V, Fernandez AF (2015). Methylation of NKG2D ligands contributes to immune system evasion in acute myeloid leukemia. Genes Immun.

[46] Paczulla AM, Rothfelder K, Raffel S (2019). Absence of NKG2D ligands defines leukaemia stem cells and mediates their immune evasion. Nature.

[47] Sallman DA, Brayer J, Sagatys EM (2018). NKG2D-based chimeric antigen receptor therapy induced remission in a relapsed/refractory acute myeloid leukemia patient. Haematologica.

[48] Driouk L, Gicobi JK, Kamihara Y (2020). Chimeric antigen receptor T cells targeting NKG2D-ligands show robust efficacy against acute myeloid leukemia and T-cell acute lymphoblastic leukemia. Front Immunol.

[49] Wu Z, Zhang H, Wu M (2021). Targeting the NKG2D/NKG2D-L axis in acute myeloid leukemia. Biomed Pharmacother.

[50] Wang Z, Guan W, Wang M (2021). AML1-ETO inhibits acute myeloid leukemia immune escape by CD48. Leuk Lymphoma.

[51] Zhang T, Fang Q, Liu P, Wang P, Feng C, Wang J (2022). Heme oxygenase 1 overexpression induces immune evasion of acute myeloid leukemia against natural killer cells by inhibiting CD48. J Transl Med.

[52] Wang Z, Xiao Y, Guan W (2020). Acute myeloid leukemia immune escape by epigenetic CD48 silencing. Clin Sci.

[53] Barakos GP, Hatzimichael E (2022). Microenvironmental features driving immune evasion in myelodysplastic syndromes and acute myeloid leukemia. Diseases.

[54] Davids MS, Kim HT, Bachireddy P (2016). Ipilimumab for patients with relapse after allogeneic transplantation. N Engl J Med.

[55] Zeidner JF, Vincent BG, Ivanova A (2021). Phase II trial of pembrolizumab after high-dose cytarabine in relapsed/refractory acute myeloid leukemia. Blood Cancer Discov.

[56] Agrawal V, Croslin C, Beltran AL (2022). Promising safety and efficacy results from an ongoing phase 1b study of pembrolizumab combined with decitabine in patients with relapsed/refractory (R/R) acute myeloid leukemia (AML). Blood.

[57] Buecklein VL, Warm M, Spiekermann K (2022). Trial in progress: an open-label phase II study of relatlimab with nivolumab in combination with 5-azacytidine for the treatment of patients with relapsed/refractory and elderly patients with newly diagnosed acute myeloid leukemia (AARON). Blood.

[58] Yang H, Bueso-Ramos C, DiNardo C (2014). Expression of PD-L1, PD-L2, PD-1 and CTLA4 in myelodysplastic syndromes is enhanced by treatment with hypomethylating agents. Leukemia.

[59] Garcia JS, Flamand Y, Penter L (2023). Ipilimumab plus decitabine for patients with MDS or AML in posttransplant or transplant-naïve settings. Blood.

[60] Penter L, Liu Y, Wolff JO (2023). Mechanisms of response and resistance to combined decitabine and ipilimumab for advanced myeloid disease. Blood.

[61] Goswami M, Gui G, Dillon LW (2022). Pembrolizumab and decitabine for refractory or relapsed acute myeloid leukemia. J Immunother Cancer.

[62] Rutella S, Vadakekolathu J, Mazziotta F (2022). Immune dysfunction signatures predict outcomes and define checkpoint blockade-unresponsive microenvironments in acute myeloid leukemia. J Clin Invest.

[63] Daver N, Garcia-Manero G, Basu S (2019). Efficacy, safety, and biomarkers of response to azacitidine and nivolumab in relapsed/refractory acute myeloid leukemia: a nonrandomized, open-label, phase II study. Cancer Discov.

[64] Radpour R, Riether C, Simillion C, Höpner S, Bruggmann R, Ochsenbein AF (2019). CD8^+^ T cells expand stem and progenitor cells in favorable but not adverse risk acute myeloid leukemia. Leukemia.

[65] Wang M, Zhang C, Tian T (2018). Increased regulatory T cells in peripheral blood of acute myeloid leukemia patients rely on tumor necrosis factor (TNF)-α-TNF receptor-2 pathway. Front Immunol.

[66] Dong Y, Han Y, Huang Y (2020). PD-L1 is expressed and promotes the expansion of regulatory T cells in acute myeloid leukemia. Front Immunol.

[67] Taghiloo S, Asgarian-Omran H (2021). Immune evasion mechanisms in acute myeloid leukemia: a focus on immune checkpoint pathways. Crit Rev Oncol Hematol.

[68] Teague RM, Kline J (2013). Immune evasion in acute myeloid leukemia: current concepts and future directions. J Immunother Cancer.

[69] Tettamanti S, Pievani A, Biondi A, Dotti G, Serafini M (2022). Catch me if you can: how AML and its niche escape immunotherapy. Leukemia.

[70] Karachaliou N, Gonzalez-Cao M, Sosa A (2017). The combination of checkpoint immunotherapy and targeted therapy in cancer. Ann Transl Med.

[71] Daver N, Alotaibi AS, Bücklein V, Subklewe M (2021). T-cell-based immunotherapy of acute myeloid leukemia: current concepts and future developments. Leukemia.

[72] Marvin-Peek J, Savani BN, Olalekan OO, Dholaria B (2022). Challenges and advances in chimeric antigen receptor therapy for acute myeloid leukemia. Cancers.

[73] Stelmach P, Trumpp A (2023). Leukemic stem cells and therapy resistance in acute myeloid leukemia. Haematologica.

[74] Rovatti PE, Gambacorta V, Lorentino F, Ciceri F, Vago L (2020). Mechanisms of leukemia immune evasion and their role in relapse after haploidentical hematopoietic cell transplantation. Front Immunol.

[75] Gournay V, Vallet N, Peux V (2022). Immune landscape after allo-HSCT: TIGIT- and CD161-expressing CD4 T cells are associated with subsequent leukemia relapse. Blood.

[76] Yan Y, Upadhyaya R, Zhang VW, Berg T (2022). Epigenetic maintenance strategies after allogeneic stem cell transplantation in acute myeloid leukemia. Exp Hematol.

